# Paternal sepsis induces alterations of the sperm methylome and dampens offspring immune responses—an animal study

**DOI:** 10.1186/s13148-018-0522-z

**Published:** 2018-06-28

**Authors:** Katharina Bomans, Judith Schenz, Sandra Tamulyte, Dominik Schaack, Markus Alexander Weigand, Florian Uhle

**Affiliations:** Department of Anesthesiology, Heidelberg University Hospital, Im Neuenheimer Feld 110, 69120 Heidelberg, Germany

**Keywords:** Intergenerational inheritance, Epigenetic, Germline, Methylation

## Abstract

**Background:**

Sepsis represents the utmost severe consequence of infection, involving a dysregulated and self-damaging immune response of the host. While different environmental exposures like chronic stress or malnutrition have been well described to reprogram the germline and subsequently offspring attributes, the intergenerational impact of sepsis as a tremendous immunological stressor has not been examined yet.

**Methods:**

Polymicrobial sepsis in 12-week-old male C57BL/6 mice was induced by cecal ligation and puncture (CLP), followed by a mating of the male survivors (or appropriate sham control animals) 6 weeks later with healthy females. Alveolar macrophages of offspring animals were isolated and stimulated with either LPS or Zymosan, and supernatant levels of TNF-α were quantified by ELISA. Furthermore, systemic cytokine response to intraperitoneally injected LPS was assessed after 24 h. Also, morphology, motility, and global DNA methylation of the sepsis survivors’ sperm was examined.

**Results:**

Comparative reduced reduction bisulfite sequencing (RRBS) of sperm revealed changes of DNA methylation (*n* = 381), most pronounced in the intergenic genome as well as within introns of developmentally relevant genes. Offspring of sepsis fathers exhibited a slight decrease in body weight, with a more pronounced weight difference in male animals (CLP vs. sham). Male descendants of sepsis fathers, but not female descendants, exhibited lower plasma concentrations of IL-6, TNF-alpha, and IL-10 24 h after injection of LPS. In line, only alveolar macrophages of male descendants of sepsis fathers produced less TNF-alpha upon Zymosan stimulation compared to sham descendants, while LPS responses kept unchanged.

**Conclusion:**

We can prove that male—but surprisingly not female—descendants of post-sepsis fathers show a dampened systemic as well as pulmonary immune response. Based on this observation of an immune hypo-responsivity, we propose that male descendants of sepsis fathers are at risk to develop fungal and bacterial infections and might benefit from therapeutic immune modulation.

**Electronic supplementary material:**

The online version of this article (10.1186/s13148-018-0522-z) contains supplementary material, which is available to authorized users.

## Background

Globally, more than 30 million people are estimated to suffer from sepsis each year [1]. Recently redefined as organ dysfunction resulting from an exaggerated systemic immune response to an underlying bacterial, fungal, or viral infection, the syndrome sepsis belongs to the ongoing challenges of modern intensive care medicine [2]. Steady improvements of treatment bundles resulted in a gradual decline in mortality over the past years, remaining anyhow on an unacceptable high level of 20–50% [3]. Sepsis not only does affect elderly persons but also can strike all ages, including patients before or within the onset of their sexual activity [4–6]. During the immune response in the early stage of sepsis, a systemic activation of immune cells leads to an uncontrolled release of cytokines, chemokines, and other mediators, e.g., reactive oxygen species [7]. The consequence is an avalanche-like forward amplifying response, impacting in its severity not only cells of the immune system but also, e.g., endothelial cells, cardiomyocytes, or skeletal muscle cells [8–10]. Furthermore, changes in progenitor cells like hematopoietic stem cells (HSC) of the bone marrow have been hypothesized to occur during inflammation [11], with recent proof of this concept during chronic inflammation in diabetes [12] as well as in the acute inflammatory condition of sepsis [13, 14]. Epigenetic mechanisms have been proposed to mediate the changes and are also found in monocytes of patients with sepsis [15]. Besides the bone marrow, also the germline contains multipotent cells, developing in males into sperm cells. While the negative impact of inflammation on the overall male fertility has been well characterized [16, 17], it is unclear if individual sperm cells or even their progenitors might also be affected on a more subtle epigenetic level, enabling the carrying forward of information onto the next generation.

Today, evidence for the non-genetic “inclusive inheritance” of a variety of environmental exposures by epigenetic and behavioral transmission to following generations is steadily growing [18, 19]. Most available evidence exists for parental exposure to different diets and emotional stress [20], with no information about the situation after severe infections or sepsis. To approach this point, we performed an animal study utilizing a clinically relevant model of polymicrobial abdominal sepsis to evaluate the transmissibility of an immunological phenotype after paternal sepsis.

## Results

### Sepsis impairs sperm function and alters the DNA methylome

**Fig. 1:**
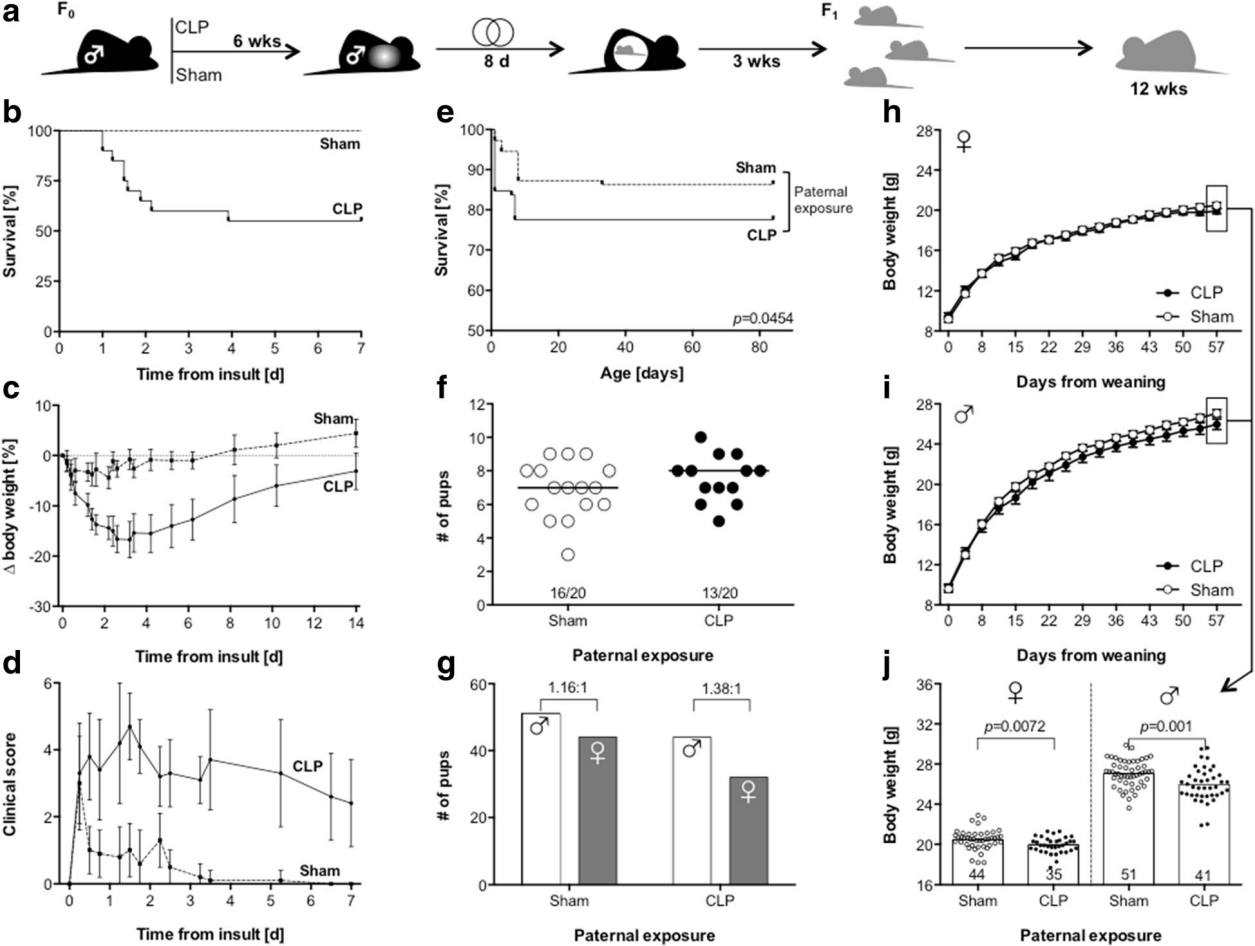
Offspring of sepsis fathers exhibit a higher mortality and altered development. **a** Experimental design of the study. **b** Survival, **c** body weight, and **d** clinical severity of CLP (*n* = 20) vs. sham (*n* = 10) operated male C57BL/6 mice of the paternal generation. **e** Survival of offspring mice (both sexes) grouped for paternal exposure (98 CLP vs. 110 sham). Group comparison was performed by Gehan-Breslow-Wilcoxon test. **f** Litter size and successful breedings (numbers). **g** Sex distribution of all litters according to paternal exposure. Body weight development of female (**h**) and male (**i**) offspring animals according to paternal exposure over 57 days after weaning, data points represent mean with 95% confidence interval. **j** Body weight on day 57 after weaning grouped for paternal exposure and sex (column height represents mean, numbers indicate sample size). Group comparison was performed by Mann-Whitney *U* test

**Fig. 2:**
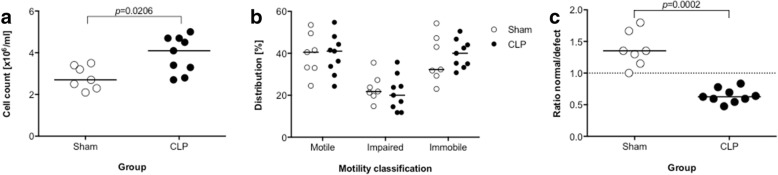
Post-sepsis animals possess more, but immobile and defect sperm. **a** Sperm cell count of individual animals assessed by swim-out from both *Caudae epidydimis* in animals after CLP (*n* = 9) or sham (*n* = 7). **b** Classification of sperm motility in both groups. **c** Ratio of morphologically normal to defect sperm in both groups. Group comparison was performed by Mann-Whitney *U* test

After CLP, nearly half of the paternal animals died within 5 days (9/20 animals), while no animal of the sham group died (Fig. 1b). The pronounced loss of body weight and higher clinical scores of CLP animals indicate the infection-associated severity of induced abdominal sepsis (Fig. 1c, d). To evaluate impact of sepsis on the paternal germline, we initially assessed sperm morphology and function. We found an increased number of sperm cells in animals after CLP (Fig. 2a), but these tended to be immobile (Fig. 2b) or to show a defect appearance (e.g., double tails or heads) (Fig. 2c).

**Fig. 3:**
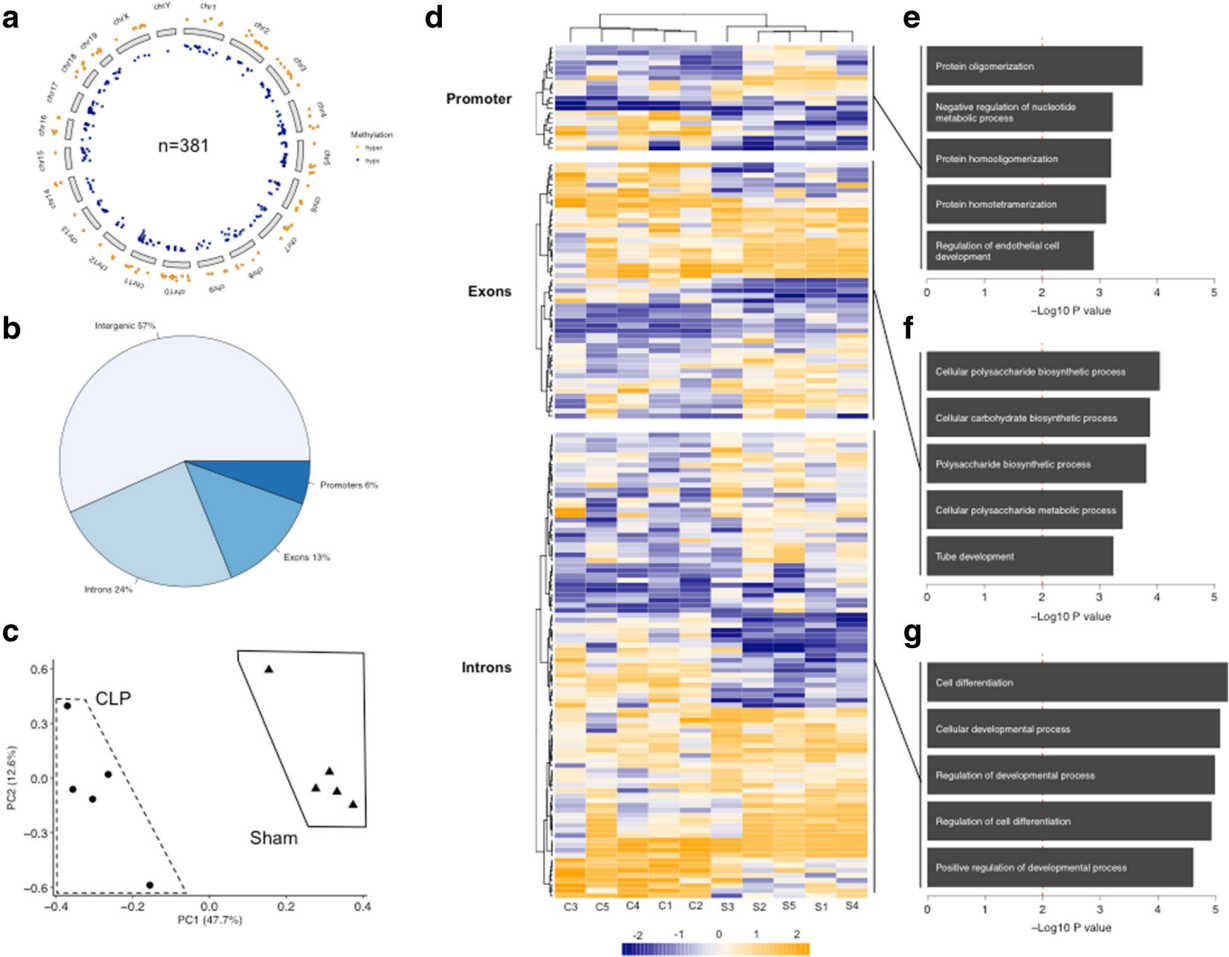
Distinct methylome alterations occur in sperm of post-sepsis animals. **a** Circos plot depicting the distribution of differentially methylated cytosines (*n* = 381) over the chromosomes (orange: hypermethylated CLP vs. sham; blue: hypomethylated CLP vs. sham). **b** Distribution of differentially methylated cytosines according to genomic region. **c** Principal component analysis using the 381 cytosines for clustering. **d** Heat map representation of differential cytosine methylation after unsupervised hierarchical clustering separated into genomic regions (promoter, exons, intron); bottom annotation represents individual animal (C = CLP, S = sham). **e**–**g** Corresponding gene lists were separately subjected to gene ontology term analysis. Top five overrepresented biological processes are shown. Dashed red line depicts statistical threshold of *p* = 0.01. Data is derived from *n* = 5 animals of each experimental condition (sham or CLP)

Our phenotypical sperm results drove us to dig deeper and to examine the sperm methylome for sepsis-induced changes. We used an independent animal cohort with comparable mortality as the mating cohort (Additional file 1: Figure S1). Six weeks after CLP, respectively after sham procedure, we isolated sperm DNA and subjected it to methylation analysis via RRBS. We can identify 381 differentially methylated cytosines (dmCs; both hypo- and hypermethylated) distributed over all chromosomes (methylation change ≥ 25%, *q* value ≤ 0.01) (Fig. 3a). The majority of dmCs was located in intergenic regions (57%) followed by introns (24%), exons (13%), and promoters (6%) (Fig. 3b). Using these 381 dmCs, the animals distinctively clustered according to their paternal exposure (Fig. 3c, d; Additional file 2: Figure S2). For dmCs located within promoter regions, exons or introns, the corresponding genes were extracted and resulting lists were separately analyzed for overrepresentation of biological functions. The results provide evidence for alterations within genes involved in metabolic, biosynthetic, and developmental processes, e.g., “regulation of endothelial cell development” (*p* = 4 × 10^−3^), “cellular carbohydrate biosynthetic process” (*p* = 1.3 × 10^−4^), and “cell differentiation” (*p* = 6.22 × 10^−6^) (Fig. 3e–g).

### Paternal sepsis increases postnatal mortality and influences development

Descendants of CLP fathers showed a significantly lower survival compared to control animals (76/98: 77.6% vs. 95/110: 86.4%; *p* = 0.0454) (Fig. 1e, Additional file 3: Table S1). The difference became evident early within the first week after birth, and with the exception of one animal (which was euthanized due to teeth malposition), no delayed deaths occurred.

Also, the number of litters, litter size, and sex distribution did not differ between the groups (Fig. 1f, g). Female and male descendants of CLP fathers both exhibited a subtle lower gain of body weight compared to descendants of control animals (Fig. 1h, i). On day 57 after weaning, the mean difference in body weight was significantly present in both genders, but more pronounced in males (25.7 vs. 27.1 g, *p =* 0.001) than in females (19.9 vs. 20.5 g, *p =* 0.0072) (Fig. 1j).

### Immunological responses are disturbed exclusively in male offspring

**Fig. 4:**
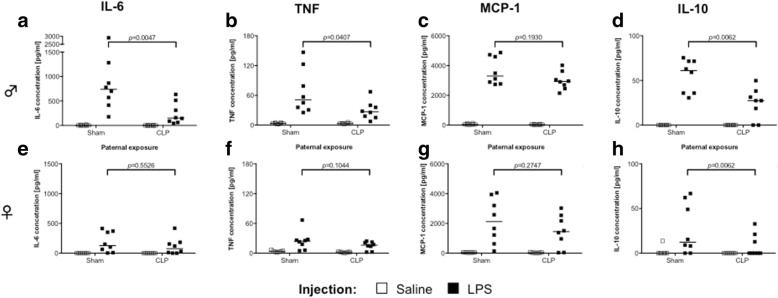
Male offspring of sepsis fathers show a reduced cytokine response after systemic LPS administration. Plasma cytokines were measured by multiplex cytometric bead array 24 h after intraperitoneal injection of saline (open squares) respectively LPS (1 mg/kg, black squares) into male (**a**–**d**) and female (**e**–**h**) offspring of fathers subjected to CLP or sham. *N* = 8 animals of each condition were used with exception of the groups of females with saline injection (*n* = 7). Horizontal line depicts the median; group comparison was performed by Mann-Whitney *U* test

We evaluated the systemic cytokine response of the animals after stimulation with the *Toll-like receptor*-4 (TLR4) agonist LPS. Males exhibited differences depending on paternal exposure, with lower pro-inflammatory IL-6 (median 151.3 vs. 738.4 pg/ml; *p =* 0.0047), TNF-α (median 26.8 vs. 51.1 pg/ml; *p =* 0.0407), and anti-inflammatory IL-10 (median 27.4 vs. 61.0 pg/ml; *p =* 0.0062) levels in the blood (Fig. 4a–d). MCP-1 levels did not differ between the paternal exposure groups. With the exception of IL-10, females did not show any response differences in respect to paternal exposure or analyzed cytokine, but generally reduced cytokine levels (Fig. 4e–h). Weight changes after LPS injection resembled these results with a trend towards a pronounced weight loss of male CLP offspring as soon as 24 h after injection of LPS (Additional file 4: Figure S3).

**Fig. 5:**
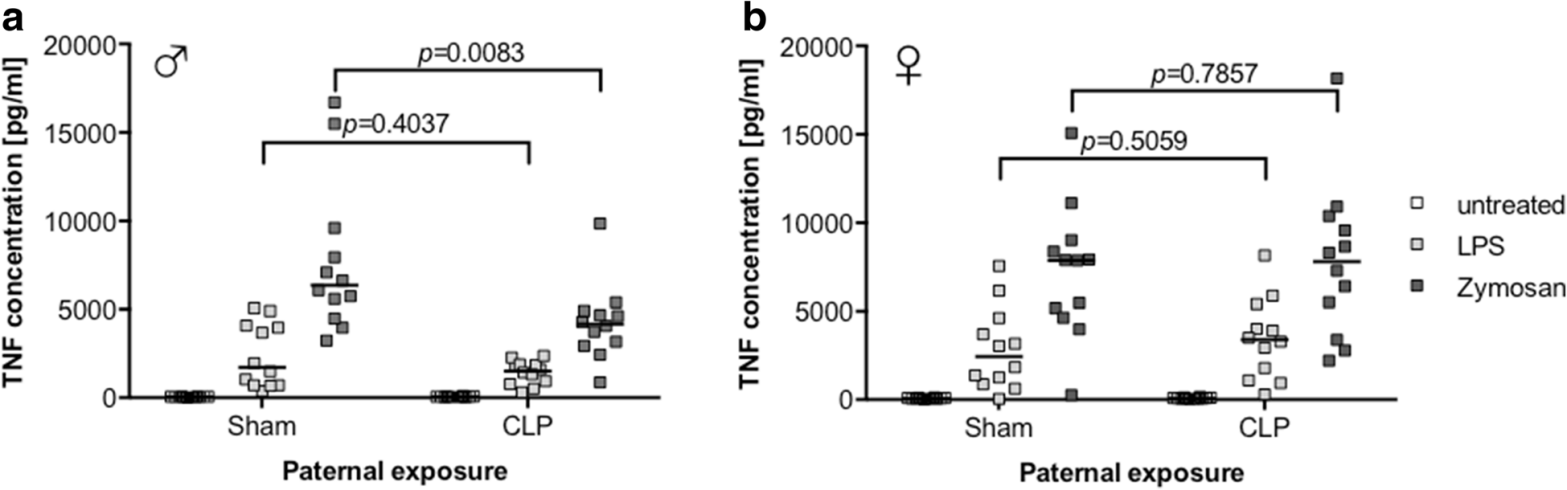
Selectively reduced response of alveolar macrophages from male offspring of sepsis fathers. TNF-α cytokine levels in the supernatant of alveolar macrophages either untreated (open squares), after stimulation with LPS (200 ng/ml; light gray squares) or zymosan (250 μg/ml; dark gray squares) of male (**a**) or female (**b**) offspring. Cells were yielded by bronchoalveolar lavage of the lungs after euthanasia; *n* = 12 for each group, horizontal line depicts median. Group comparisons were performed by Mann-Whitney *U* test

Furthermore, we tested the response of isolated alveolar macrophages stimulated with either LPS or the Dectin-1 agonist zymosan. Alveolar macrophages from male CLP offspring showed a reduced TNF-α response to zymosan compared to controls (median 4182 vs. 6357 pg/ml, *p =* 0.0083), while the response to LPS was not affected (median 1510 vs. 1719 pg/ml) (Fig. 5a). In contrast, the same experiment conducted in alveolar macrophages isolated from female offspring showed no difference in response, irrespective of the stimulus (Fig. 5b).

## Discussion

Using a polymicrobial animal model of abdominal sepsis, our results are the first to our knowledge providing evidence for an intergenerational transmission of immunological changes from fathers to their male, but apparently not female, offspring. We further propose the sperm methylome as a potential carrier of information between the generations, as we can show differentially hypo- and hypermethylation of cytosine residues spread over the genome. The mechanisms of epigenetic inheritance through the male germline are still under debate, especially considering the replacement of histones to protamine during spermatozoa development and the global erasure of DNA methylation after fertilization [20]. Nevertheless, several studies involving paternal (mal-)nutritional exposures as, e.g., high-fat, low-protein, or low-folate [21–23] or the exposure to environmental toxicants [24, 25] clearly indicate a crucial role of changes in the sperm methylome. As a mechanistic rationale, Guibert and colleagues provided evidence that certain methylated cytosines “escape” the TET-mediated wave of demethylation during the development of primordial germ cells [26, 27]. Besides, DNA methylation of disease-associated loci in sperms changes with age, and the association of impaired DNA methylation with male infertility has been proven in several studies so far [28, 29]. Interestingly, we can recapitulate this concept in our animal model of sepsis with more immobile sperm and the occurrence of morphologically aberrant sperm cells even 6 weeks after the insult. In contrast, survivors of sepsis exhibited more sperm cells, hinting towards a compensatory mechanism. This finding is in line with results of Kajihara et al., which used bacterial LPS to induce testicular dysfunction in mice and found this phenomenon to happen transiently after 1 week due to apoptotic cell death followed by a prolonged overcompensation of sperm production up to 5 weeks [30]. Despite the observed epigenetic and morphological alterations, the overall reproductive performance of the male survivors in our study was not impaired. Considering the time since septic insult in respect to duration of murine spermiogenesis [31], we can exclude that the sperm cells present at mating have been exposed themselves to the systemic inflammation or its mediators, hinting towards a long-lasting impact of inflammation on the spermatogonia precursors.

Leaping forward, the impact of these alterations were obvious in the early postnatal stage, with a higher loss of animals occurring in sepsis offspring than control offspring. This result might be driven by a maternal rejection of or aggression behavior against the pups, maybe as a consequence of poor offspring health. The differential weight gain of surviving pups after weaning points towards a developmental genetic trait carried forward from the fathers. In line with this, the methylation changes in intronic and promoter regions of genes associated with developmental processes have been found overrepresented, besides alterations of cytosine methylation within promoters and exons of genes involved in metabolism. Strong evidence for the intergenerational impact of subtle alterations of the sperm methylome on offspring’s metabolic function has been provided recently [32]. This study used in utero undernourishment as metabolic stressor and found metabolic alterations in the descendants. Comparably, during the acute phase of abdominal sepsis, the mice transiently lost substantial weight due to a sickness behavior-associated lower food intake aggravated by the high metabolic demand of the activated immune system, closely mimicking the clinical condition. Besides the abovementioned, several studies report inter- and transgenerational heredity of parental under- and overnutrition as a genetic trait for offspring’s metabolism and body weight, making it a potential contributing factor also in our study [22, 33–36]. Alternatively, other studies involving animals and human subjects prove the transgenerational inheritance of experienced environmental stress (e.g., noise) [37–39]. With sepsis resembling an enormous stressful condition involving the excessive activation of, e.g., the neuroendocrine hypothalamic–pituitary–adrenal axis [35, 40], these might also adaptively lower the offspring’s stress response threshold, thereby again increasing basal energy consumption and lowering body weight. Also, emerging evidence shows the importance of different cellular metabolic pathways as fundamental determinants of immune cell function in sepsis and other conditions [41, 42].

We report here a loss of immunological responsivity of male descendants of sepsis fathers upon intraperitoneal LPS injection, indicated by reduced plasma cytokines. We can neither rule out functional or numerical alterations in immune cell populations in the peritoneal cavity nor in the circulation as underlying reason for the observed phenotype. However, considering the fact that alveolar macrophages from our male mice showed comparably reduced, but yet stimuli dependent, responses might underline the conceptual framework of germline transmission and cellular reprogramming, happening already in the development of embryonic tissues of immunological relevance (e.g., the bone marrow or fetal liver). The lungs of adult mice are populated by macrophages originating from different tissue sources, with Mac2 expressing macrophages from fetal liver being the dominating and self-renewing population within the alveoli and therefore in our experimental design [43]. Alternatively, recent work from Roquilly et al. was able to prove that the lung microenvironment after infection induces anergy of dendritic cells by a regulatory T cell dependent and TGF-β-mediated mechanism [44]. How the specific reduction of the response to zymosan, a fungal cell wall component, but not bacterial LPS, is mediated remains elusive. Taken together, our current results hint towards a predisposing phenotype for invasive fungal infections, especially at the lung barrier.

Our findings might be interpreted in two ways: either the observed F_1_ phenotype is a “protective burden” of host tolerance to evade excessive inflammation as experienced by the fathers or it represents a compensatory maladaptive state, rendering male descendants more susceptible to microbial encounters. An example for the latter has been shown in an experimental model of paternal chronic colitis, in which offspring exhibited an increased susceptibility and disease severity [45].

Considering the phenotype of lower body weight shared from both genders, it seems to be obvious that our immunological findings must result from a synergistic interaction of the transmitted epimutations and the well-known gender dimorphism of immune responses [46]. Also, other groups showed sex-specific intergenerational phenotypes: Sanchez-Garido and colleagues proved a pronounced impact of paternal obesity on male offspring, explained by hormonal synergism [47]. In contrast, Ng et al. found paternal high-fat diet to exclusively reprogram female offspring’s pancreatic beta cells [48]. Nevertheless, especially in respect to the observed weight phenotype, we cannot rule out that when applying other immunological challenges (e.g., infection or autoimmune models), female offspring might uncover changes in responsivity and vulnerability as well.

As a mechanistical framework, we propose that paternal sepsis alters spermatogonia progenitors, which propagate their acquired epigenetic changes through meiosis into the mature sperm methylome. This information is then carried over into the zygote. Earlier studies in zebrafish prove that the sperm methylome is in fact inherited and even dominates over the maternal methylome during early embryonic development [49, 50], potentially assisted by the layer of histones [51]. With changes of the methylome present in the early embryo, the stage is set for the further propagation into all developing tissues, including the seeding immune cells. Further studies need to unravel how the identified changes are maintained through embryogenesis and how it penetrates and modulates offspring’s post-natal development and immune function. Furthermore, research involving patients in the reproductive age after sepsis is necessary to prove the translation of this concept to the post-clinical setting in regard to fertility and molecular alterations of sperm cells. From a methodical point, we just measured the tip of the iceberg, as RRBS only covers 5–10% of the genome’s cytosine content and a tremendous amount of information might be still hidden, extractable by single-base-resolution sequencing methods.

In summary, we can provide evidence for sepsis-induced epigenetic changes of the sperm methylome as a hub of non-sequence coded intergenerational information transfer, shaping offsprings’ development and immune competence.

## Conclusion

While the incidence of sepsis is steadily rising, modern high-performance medicine increases the number of surviving patients. Our results of epigenetically transmitted, intergenerational immunological changes in the offspring of sepsis sires open a whole new perspective, driving the necessity to undertake further research measures to understand the post-sepsis challenges.

## Methods

### Study approval

All animal procedures were conducted in accordance with the German Protection of Animals Act law and were approved by the regional council Karlsruhe (reference number G-132/15).

### CLP sepsis model

Male C57BL/6 mice aged 10–12 weeks were obtained from Janvier Laboratories (Le Genest Saint Isle, France). Mice were housed in a 12-h light/dark cycle at 22 °C. Food and water were provided ad libidum. Animals were allowed to acclimatize for 7 days before any experimental procedure. Polymicrobial sepsis was induced using the cecal ligation and puncture (CLP) model [52]. In total, 20 mice were anesthetized with 100 mg/kg ketamine (Ketanest®S, Pfizer Pharma, Berlin, Germany) and 20 mg/kg xylazine (Xylavet, CP-Pharma, Burgdorf, Germany) intraperitoneally. After a midline laparotomy and mobilization of the cecum, 5 mm was ligated and punctured once with a 23-G needle (BD Microlance™ 3, BD Medical, Heidelberg, Germany). The ligated cecum was pressed gently to extrude fecal contents. Afterwards, the cecum was relocated, the mice were supplemented with 400 μl 0.9% NaCl (B. Braun, Melsungen, Germany), given directly into the abdominal region, and the abdomen was closed with a double suture. For control, 10 animals underwent a laparotomy surgery only applying the same anesthesia regime. After surgery, pain relieve in both groups was achieved by treatment with 0.05 mg/kg bodyweight buprenorphine (Temgesic, RB Pharmaceuticals, Slough, UK) every 8 h for 2 days. For the isolation and analysis of sperm, a separate cohort of animals, both CLP as well as sham, was operated.

#### Sperm isolation and analysis

Mature sperm cells were isolated from the Cauda epididymis of 9 CLP and 7 sham mice into Donners medium (135 mM NaCl (Sigma-Aldrich, Steinheim, Germany), 5 mM KCl (Merck, Darmstadt, Germany), 1 mM MgSO_4_ (Sigma-Aldrich, Steinheim, Germany), 2 mM CaCl_2_ (Sigma-Aldrich, Steinheim, Germany), 30 mM HEPES pH 7.4 (Roth, Karlsruhe, Germany); freshly supplemented with 0.53% sodium lactate (Caelo, Hilden, Germany), 1 mM sodium pyruvate (Life Technologies, Darmstadt, Germany), 20 mg/mL BSA (Roth, Karlsruhe, Germany) and 25 mM NaHCO_3_ (Roth, Karlsruhe, Germany)) at 37 °C and 5% CO_2_ for 40 min via swim-up assay. To avoid contamination with somatic cells, only the top fractions were used for further examinations.

Sperm motility, morphology, and total cell numbers from CLP and sham mice were assessed according to WHO guidelines [53]. Total cell numbers were counted using a hemocytometer. For both, sperm motility and morphology, 200 cells of each isolation were analyzed (in replicate) using a Keyence Biozero microscope (Keyence, Neu-Isenburg, Germany). Sperm motility was scaled into three categories: progressive motility (PR), non-progressive motility (NP), and immotile (IM). Cell-VU® Prestained Morphology Slides (Millenium Sciences, NY, USA) were used according to the manufacturer’s protocol for proper visualization. Sperm cells morphology was judged as normal or defect.

Details on DNA extraction, sequencing, and bioinformatics are given in Additional file 3: Document S1.

### Breeding scheme and characterization of litters

For breeding, all CLP survivors and sham mice were used. Each male was mated with two infection-naive C57BL/6 females (aged 12 weeks) 6 weeks after induction of sepsis (Fig. 1a). To exclude paternal effects on maternal care and subsequently offspring survival as well as development, males were separated after 8 days resembling two full estrous cycles [23].

After birth, litter size was determined and survival of pups assessed over 12 weeks while held under standard housing conditions within the same facility. Pregnancy occurred in 13 of 20 CLP breeding pairs and 16 of 20 sham breeding groups. Maternal weaning and offspring sex determination occurred on day 23 postnatally. For the investigation of weight development, offspring were weighted two times per week for a total of 57 days after weaning.

### TNF-α levels of alveolar macrophages in offspring

Alveolar macrophages were isolated via bronchoalveolar lavage (BAL) in 12-week-old animals. Offspring from CLP survivors and control sires (*n* = 12 for each sex and parental exposure) were euthanized by bleeding under ketanest/xylazin anesthesia, and BAL was performed by flushing the lung with 1 ml ice-cold phosphate-buffered saline (PBS) (Life Technologies, Umkirch, Germany) for 10 times through an incision of the trachea. Cells were counted, and 1 × 10^6^ cells were seeded in a 96-well plate (Sarstedt, Nuembrecht, Germany) with Aim V Media (Life Technologies, Darmstadt, Germany). Stimulation was done with 200 ng/ml ultrapure LPS or 250 μg/ml depleted Zymosan (both Invivogen, Toulouse, France). No stimulating agent was added to controls. After incubation for 24 h (37 °C with 5% CO_2_), supernatant was collected and TNF-α levels were determined using Mouse TNF-alpha DuoSet ELISA (R&D Systems, Minneapolis, USA) according to the manufacturer’s instruction. In case of low BAL cell yield, lower cell numbers were used for stimulation and response was normalized accordingly.

### In vivo stimulation and cytokine analysis of offspring

Offspring from CLP survivors and sham sires (aged 32 weeks) were injected with LPS (1 mg/kg). For each group, eight animals were used except for saline-treated female groups (*n* = 7) After 24 h, mice were euthanized and the blood was collected by cardiac puncture. Mice were weighted before injection as well as before euthanasia.

For flow cytometry-based multiplex cytokine analysis, the CBA Mouse Inflammation Kit (BD Biosciences, Heidelberg, Germany) was used according to the manufacturer’s protocol. Detection was performed with a BD FACSVerse flow cytometer and data analyzed by FCAP Array 3.0 software (BD Biosciences, Heidelberg, Germany).

### Statistics

All statistical analysis and visualizations were done in GraphPad Prism (V6.0 for Mac, GraphPad Software, La Jolla, USA). Comparisons between two groups were performed depending on the sample size and distribution either by non-parametric Mann-Whitney *U* test (two-tailed; for non-normal distribution and *n* ≤ 30) or unpaired *t* test (two-tailed; normally distributed data or *n* > 30). Survival time analysis was done by applying the Gehan-Breslow-Wilcoxon test to take into account the occurrence of early events over the observation time. Statistical significance was assumed with a *p* value of less than 0.05 for all analysis.

## Additional files

Additional file 1: **Figure S1.** Survival of the second animal cohort used for sperm analysis with CLP (*n* = 15) vs. sham (*n* = 9) male C57BL/6 mice. (TIFF 3074 kb)

Additional file 2: **Figure S2.** Heat map representation of differential cytosine methylation in intergenic regions after unsupervised hierarchical clustering. Bottom annotation represents individual animal (C = CLP, S = sham). (TIFF 3074 kb)

Additional file 3: **Document S1.** Extended methods including sperm DNA isolation, sequencing and bioinformatic analysis. (DOCX 27 kb)

Additional file 4: **Figure S3.** Relative weight loss of animals 24 h after intraperitoneal LPS injection (1 mg/kg). Female (A) or male (B) offspring of both control (“sham”) and post-sepsis (“CLP”) fathers were weighted before and 24 h after injection, and percentage weight change was calculated. *N* = 8 for each group except female groups with saline injection (*n* = 7). Solid horizontal lines depict median, dashed line equals zero change. (TIFF 3074 kb)

## Abbreviations

CLP: Cecal ligation and puncture
dmCs: Differentially methylated cytosines
DNA: Deoxyribonucleic acid
HSC: Hematopoietic stem cells
IL-6/-10: Interleukin-6/-10
LPS: Lipopolysaccharide
MCP-1: Monocyte chemoattractant protein 1
RRBS: Reduced reduction bisulfite sequencing
TGF-β: Transforming growth factor β
TLR4: Toll-like receptor 4
TNF-α: Tumor necrosis factor α
