# Supplementary figures and images for: Paternal sepsis induces alterations of the sperm methylome and dampens offspring immune responses—an animal study
Source: Clin Epigenetics. 2018 Jun 28;10:89. doi: 10.1186/s13148-018-0522-z (PMC6022485; doi:10.1186/s13148-018-0522-z)

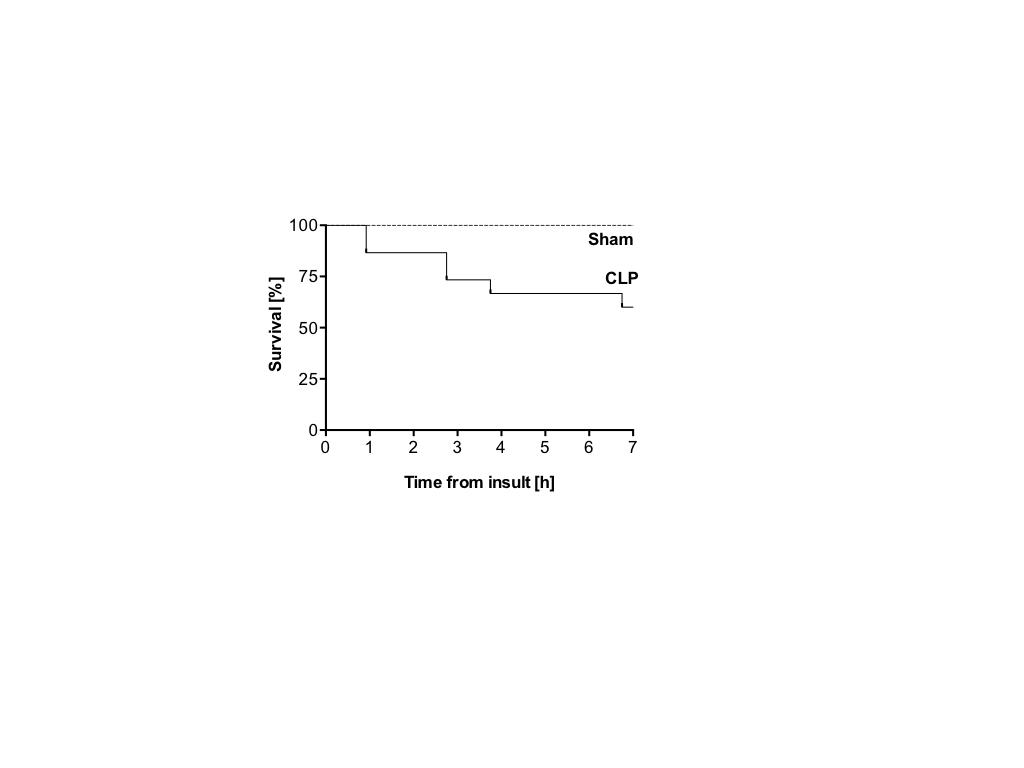

Supplement: Supplementary file 1 — Figure S1. Survival of the second animal cohort used for sperm analysis with CLP (n = 15) vs. sham (n = 9) male C57BL/6 mice. (TIFF 3074 kb) [file 13148_2018_522_MOESM1_ESM.tiff]

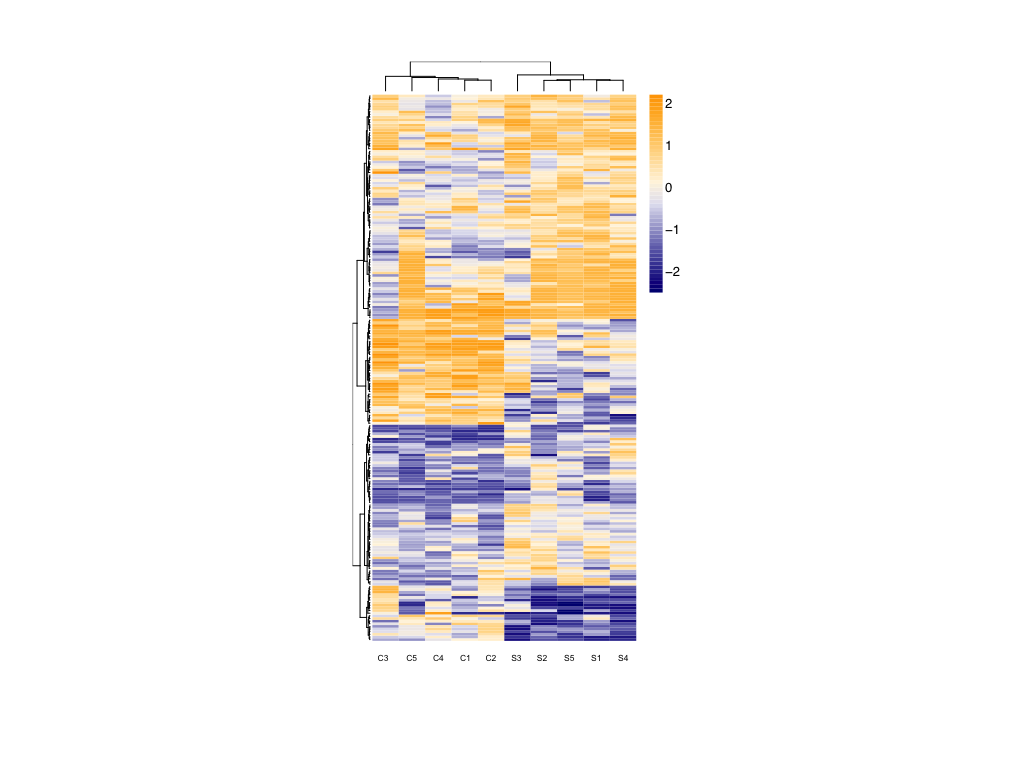

Supplement: Supplementary file 2 — Figure S2. Heat map representation of differential cytosine methylation in intergenic regions after unsupervised hierarchical clustering. Bottom annotation represents individual animal (C = CLP, S = sham). (TIFF 3074 kb) [file 13148_2018_522_MOESM2_ESM.tiff]

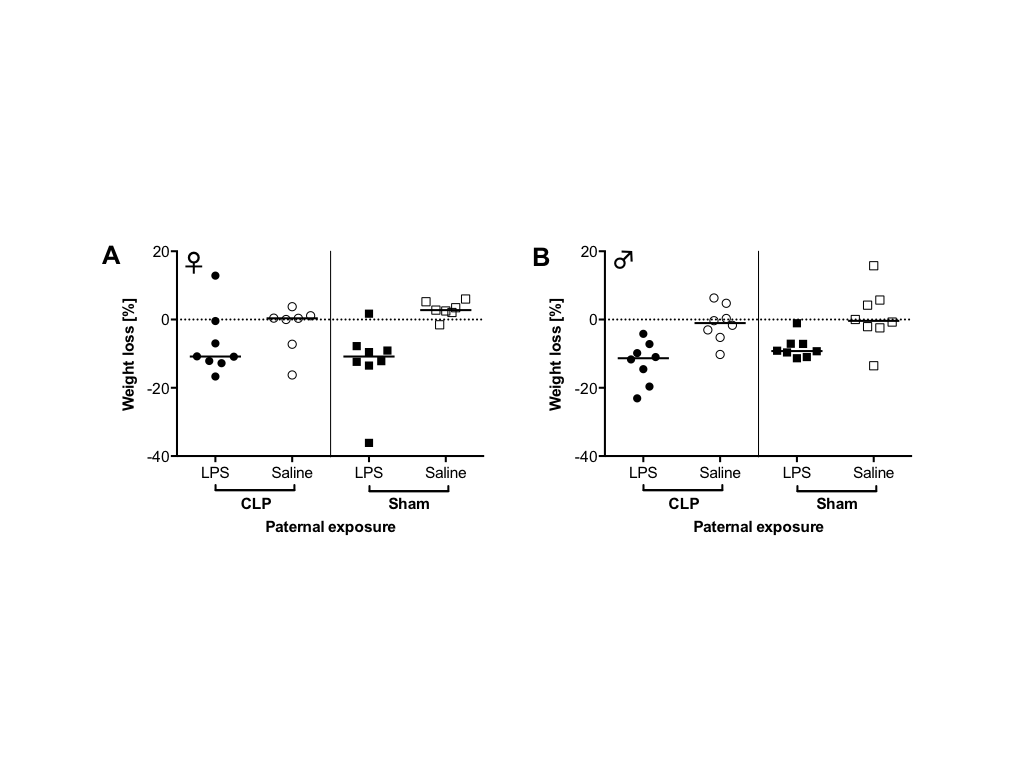

Supplement: Supplementary file 4 — Figure S3. Relative weight loss of animals 24 h after intraperitoneal LPS injection (1 mg/kg). Female (A) or male (B) offspring of both control (“sham”) and post-sepsis (“CLP”) fathers were weighted before and 24 h after injection, and percentage weight change was calculated. N = 8 for each group except female groups with saline injection (n = 7). Solid horizontal lines depict median, dashed line equals zero change. (TIFF 3074 kb) [file 13148_2018_522_MOESM4_ESM.tiff]
