# Supplementary material for: Paternal sepsis induces alterations of the sperm methylome and dampens offspring immune responses—an animal study
Source: Clin Epigenetics. 2018 Jun 28;10:89. doi: 10.1186/s13148-018-0522-z (PMC6022485; doi:10.1186/s13148-018-0522-z)
Supplement: Supplementary file 3 — Document S1. Extended methods including sperm DNA isolation, sequencing and bioinformatic analysis. (DOCX 27 kb) [file 13148_2018_522_MOESM3_ESM.docx]

**Supplementary information**

Sperm isolation and analysis

Mature sperm cells were isolated from the Cauda epididymis of 9 CLP and 7 Sham mice into Donners medium (135 mM NaCl (Sigma Aldrich, Steinheim, Germany), 5 mM KCl (Merck, Darmstadt, Germany), 1 mM MgSO_4_ (Sigma Aldrich, Steinheim, Germany), 2 mM CaCl_2_ (Sigma Aldrich, Steinheim, Germany), 30 mM HEPES pH7.4 (Roth, Karlsruhe, Germany); freshly supplemented with 0.53% sodium lactate (Caelo, Hilden, Germany), 1 mM sodium pyruvate (Life Technologies, Darmstadt, Germany), 20 mg/mL BSA (Roth, Karlsruhe, Germany) and 25 mM NaHCO_3_ (Roth, Karlsruhe, Germany)) at 37°C and 5% CO_2_ for 40 min via swim-up assay. To avoid contamination with somatic cells, only the top fractions were used for further examinations.

Sperm motility, morphology and total cell numbers from CLP and Sham mice were assessed according to WHO guidelines (1). Total cell numbers were counted using a hemocytometer. For both, sperm motility and morphology, 200 cells of each isolation were analyzed (in replicate) using a Keyence Biozero microscope (Keyence, Neu-Isenburg, Germany). Sperm motility was scaled into three categories: Progressive motility (PR), non-progressive motility (NP) and immotile (IM). Cell-VU® Prestained Morphology Slides (Millenium Sciences, New York, USA) were used according to the manufacturer’s protocol for proper visualization. Sperm cells morphology was judged as normal or defect.

Details on DNA extraction, sequencing and bioinformatics are given in the Supplementary informations.

DNA isolation from sperm

DNA extraction was performed using the Qiagen DNA Blood Mini Kit (Qiagen, Hilden, Germany) in modified form (2). 10^6^ sperm cells from CLP or Sham animals were treated with somatic cell lysis buffer (0.1% SDS (Roth, Karlsruhe, Germany), 0.5% Triton X (Merck, Darmstadt, Germany) in ddH2O) for 10 min on ice. Cells were washed twice, resuspended in 500 µl RLT with DTT (150 mM, Roche Diagnostics, Rotkreuz, Switzerland) and homogenized using Precellys MK28 steel beads with a Precellys® Evolution homogenizer (Bertin Technologies, Montigny-le-Bretonneux, France). Lysates were mixed with equal volumes of Buffer AL and 100% ethanol and loaded onto the spin columns. Further steps followed the manufacturer’s protocol.

DNA isolate was concentrated using the Genomic DNA Clean and Concentrator-10 Kit (Zymo Research, Irvine, USA) according to manufacturer’s instructions. Quality was determined using the Nanodrop spectrophotometer and quantity measured with a Qubit fluorometer (Thermo Scientific, Dreieich, Germany). Finally, five DNA samples from CLP as well as Sham control animals were used for RRBS analysis.

Analysis of sperm methylome

Sperm DNA from 5 animals of each exposure group (CLP and Sham) was subjected to Reduced Reduction Bisulfite Sequencing (RRBS) (service from Diagenode s.s., Liege, Belgium) as described earlier (3). In short, DNA was digested with the restriction enzyme *MspI* and after library preparation the samples were sequenced on an Illumina HiSeq 3000.

Quality control and adapter trimming for all samples was carried out using "Trim Galore!" software, which was run in *-RRBS* mode. The default Phred score of 20 was used as the quality cut-off value. For trimming the required overlap with adapter sequence (stringency) parameter was set to a value of “1”, while maximum error rate was defined as “0.1”. Minimum read length was defined as 15bp. Quality control of data was undertaken using “FastQC” software. Sequences were mapped using single end mapping to bisulfite converted mouse genome (mm10) using “Bismark” (4) and utilizing the “Bowtie2” short read aligner (5). Calculation of DNA methylation levels and per base methylation calls were performed subsequently in the “Bismark” pipeline. Downstream processing and visualization was performed in R/Bioconductor environment using packages "methylKit", "GenomicFeatures", "annotatr", "ggfortify", "NMF" and "ggbio" (6-9). For differential statistics, minimum methylation difference cut-off was defined as 25% with a q-value of ≤ 0.01. Annotation of differentially methylated CpGs is based on mm10 refGene and CpG island annotation from UCSC (10). GO term analyses were done using GeneRanker (11) run on a Genomatix Genome Analyzer (Genomatix, Munich, Germany).

*Breeding scheme and characterization of litters*

For breeding, all CLP survivors and sham mice were used. Each male was mated with two infection-naive C57BL/6 females (aged 12 weeks) six weeks after induction of sepsis (**Supplementary Figure 1A**). To exclude paternal effects on maternal care and subsequently offspring survival as well as development, males were separated after eight days resembling two full estrous cycles (12).

After birth, litter size was determined and survival of pups assessed over 12 weeks while held under standard housing conditions within the same facility. Pregnancy occurred in 13 of 20 CLP breeding pairs and 16 of 20 Sham breeding groups. Maternal weaning and offspring sex determination occurred on day 23 postnatally. For the investigation of weight development, offspring were weighted two times per week for a total of 57 days after weaning.

**Tables**

**Table S1** Numbers of *animal-at-risk* over time (as visualized in Fig. 1E). Day 1 represents day of birth.

|  | **Day** | **1** | **3** | **6** | **7** | **8** | **33** | **84** |
| --- | --- | --- | --- | --- | --- | --- | --- | --- |
| ***Animals-at-risk*** | Sham | 110 | 107 | 107 | 107 | 104 | 96 | 95 |
|  | CLP | 98 | 98 | 83 | 82 | 82 | 82 | 76 |

**References**

1. World Health Organization: WHO Laboratory Manual for the Examination and Processing of Human Semen. 2010.

2. Wu H, de Gannes MK, Luchetti G, et al.: Rapid method for the isolation of mammalian sperm DNA. *BioTechniques* 2015; 58:293–300

3. Meissner A, Gnirke A, Bell GW, et al.: Reduced representation bisulfite sequencing for comparative high-resolution DNA methylation analysis. *Nucleic Acids Res* 2005; 33:5868–5877

4. Krueger F, Andrews SR: Bismark: a flexible aligner and methylation caller for Bisulfite-Seq applications. *Bioinformatics* 2011; 27:1571–1572

5. Langmead B, Salzberg SL: Fast gapped-read alignment with Bowtie 2. *Nat Methods* 2012; 9:357–359

6. Akalin A, Kormaksson M, Li S, et al.: methylKit: a comprehensive R package for the analysis of genome-wide DNA methylation profiles. *Genome Biol* 2012; 13:R87

7. Lawrence M, Huber W, Pagès H, et al.: Software for computing and annotating genomic ranges. *PLoS Comput Biol* 2013; 9:e1003118

8. Gaujoux R, Seoighe C: A flexible R package for nonnegative matrix factorization. *BMC Bioinformatics* 2010; 11:367

9. Yin T, Cook D, Lawrence M: ggbio: an R package for extending the grammar of graphics for genomic data. *Genome Biol* 2012; 13:R77

10. Rosenbloom KR, Armstrong J, Barber GP, et al.: The UCSC Genome Browser database: 2015 update. *Nucleic Acids Res* 2015; 43:D670–81

11. Gonzalez G, Uribe JC, Armstrong B, et al.: GeneRanker: An Online System for Predicting Gene-Disease Associations for Translational Research. *Summit Transl Bioinform* 2008; 2008:26–30

12. Rodgers AB, Morgan CP, Bronson SL, et al.: Paternal stress exposure alters sperm microRNA content and reprograms offspring HPA stress axis regulation. *J Neurosci* 2013; 33:9003–9012
